# Trajectories of Quality of Life among Chinese Patients Diagnosed with Nasopharynegeal Cancer

**DOI:** 10.1371/journal.pone.0044022

**Published:** 2012-09-18

**Authors:** Wendy W. T. Lam, Michelle Ye, Richard Fielding

**Affiliations:** Centre for Psycho-oncology Research and Teaching, School of Public Health, The University of Hong Kong, Hong Kong, People's Republic of China; University of Pennsylvania, United States of America

## Abstract

**Objective:**

This secondary longitudinal analysis describes distinct quality of life trajectories during eight months of radiation therapy (RT) among patients with nasopharyngeal cancer (NPC) and examines factors differentiating these trajectories.

**Methods:**

253 Chinese patients with NPC scheduled for RT were assessed at pre-treatment, and 4 months and 8 months later on QoL (Chinese version of the FACT-G), optimism, pain, eating function, and patient satisfaction. Latent growth mixture modelling identified different trajectories within each of four QoL domains: Physical, Emotional, Social/family, and Functional well-being. Multinomial logistic regression compared optimism, pain, eating function, and patient satisfaction by trajectories adjusted for demographic and medical characteristics.

**Results:**

We identified three distinct trajectories for physical and emotional QoL domains, four trajectories for social/family, and two trajectories for functional domains. Within each domain most patients (physical (77%), emotional (85%), social/family (55%) and functional (63%)) experienced relatively stable high levels of well-being over the 8-month period. Different Physical trajectory patterns were predicted by pain and optimism, whereas for Emotion-domain trajectories pain, optimism, eating enjoyment, patient satisfaction with information, and gender were predictive. Age, appetite, optimism, martial status, and household income predicted Social/family trajectories; household income, eating enjoyment, optimism, and patient satisfaction with information predicted Functional trajectories.

**Conclusion:**

Most patients with NPC showed high stable QoL during radiotherapy. Optimism predicted good QoL. Symptom impacts varied by QoL domain. Information satisfaction was protective in emotional and functional well-being, reflecting the importance in helping patients to establish a realistic expectation of treatment impacts.

## Introduction

Nasopharyngeal carcinoma (NPC) is a significant cancer predominating in certain populations and ethnic groups originating from South-East Asia and Southern China, Polynesia, Southern Africa, the Middle-East and North Africa, and the Arctic [1]–[2]. The disease has been linked to viral, diet, smoking and genetic factors but remains poorly understood [1]. There were an estimated 84,000 incident cases and 56,000 deaths attributable to NPC in 2008 [2]. Whist constituting only 0.7% of the world cancer burden [2], in those countries most affected NPC is a significant disease. In southeast Asia it constitutes up to the sixth most common cancer in countries where a high population proportion is of Southern Chinese descent [2], so much so that it is often referred to colloquially as “the Chinese cancer”. Incidence is approximately three times higher among males than females [1]–[2].

A cancer diagnosis carries significant implications, is highly stressful, and often generates a range of physical and psychosocial sequelae affecting patients' quality of life (QoL). QoL is an important parameter in cancer management; it is commonly used to evaluate treatment side-effects on patients' physical, psychological, and social functioning [3]. External radiotherapy with or without chemotherapy is the primary treatment for NPC. The usual radiation dose to the primary tumor is around 70 Gy in 7 weeks, which can cause various post-irradiation side effects including hearing loss, CNS damage, salivary gland dysfunction (xerostomia), altered taste and dental deterioration producing eating difficulties (dysphagia), chronic sinonasal symptoms, and neck stiffness [4]–[5]. The impacts on patients' QoL of NPC diagnosis and treatments have been extensively documented in cross-sectional studies compared to controls [6]–[9] and a smaller number of longitudinal studies [10]–[13]. Symptom load [6]–[9], particularly dysphagia [6]–[9], [14] correlates with QoL, with optimism mediating between dysphagia and QoL [12] whilst satisfaction with care is linked to better QoL [15]. Previous prospective studies suggest that QoL improves progressively throughout the first year following the diagnosis of NPC [11], [13]. However, because most longitudinal studies currently use averaged group data to examine changes over time distinctive individual or sub-group variation in patterns of QoL change that might signal amenable clinical need is hidden. Two consequences of averaging group data on adaptation are, first that the current “averaged-data” literature implies that cancer patients are initially very distressed on diagnosis and throughout treatment but gradually this distress declines. The second consequence is the widespread assumption that distress is a universal response to a cancer diagnosis. Consequently support services for all cancer patients, including screening for distress, are being widely implemented. However, evidence is accumulating that distress is neither universal nor follows a uniform trajectory from high to low over time [16], [17]. Costs make providing support services for all cancer patients a potentially expensive proposition. However, if most cancer patients are not distressed, or only transiently so, then what limited resources there are for supporting patients would be best targeted at those most in need.

Bonanno has proposed four distinct patterns of adjustment in response to potential trauma: chronic disruption of normal functioning, recovery with a relatively mild and short-lived disruption of functioning, delayed disruption of functioning, and resilience with little or no disruption of functioning [18]. Resilience is considered to be the most common outcome in response to potential trauma. This conception has been tested on patients in response to the diagnosis of cancer [16], [17], [19]–[22]. Consistent with Bonanno's postulation, cancer patients demonstrate patterns of adjustment over time that feature distinct trajectories of change in adjustment following cancer diagnosis [16], [17], [19]–[22], with most cancer patients reporting little functional disruption, smaller numbers having initial functional disruption that remits or subsequent transient disruptions, and a minority who experience persistent chronic disruption of normal functioning. For instance, among Chinese women diagnosed with breast cancer [16] distinct distress trajectories exist, with around 15% of women evidencing persistently high levels of distress (Chronic), 12% had high-to-low declining distress trajectories (Recovered) and 7% had low-high-low distress trajectories (Delayed-recovered). However, in contrast with the prevailing view from averaged data studies that most women are significantly distressed during breast cancer, a majority of these women (66%) showed persistently low and stable levels of distress throughout the post-surgical period [16]. Similarly, four distinct trajectories were identified in a sample of American women with breast cancer, though using the SF-36 to measure QoL mental and physical component scale proportions differed compared to those reported in Chinese women [20]. Nonetheless, most of these American women, like their Chinese counterparts were in the highest functioning trajectory that showed little change over time. Similar distinct trajectory patterns have been reported in cancer patients with advanced disease [19] and those receiving radiation therapy [21]. However, to date most trajectory studies focus on women with breast cancer. Other cancers have rarely been examined this way. In NCP existing longitudinal studies of QoL have also used averaged data from all patients, thereby obscuring individual patterns of change.

To address this gap we report a secondary analysis of an existing longitudinal dataset to explore adjustment trajectories in QoL over three time points before, during and on completion of eight-months of radiation therapy among patients with NPC. We also examined factors that might differentiate distinct QoL trajectories. Based on previous research [11], [12], [14]–[17], we hypothesized a priori that three distinct influences might differentiate distinct trajectories. First, dispositional optimism was hypothesized to enhance QoL because consistent evidence showed optimism is protective against psychological deterioration during cancer, suggesting optimists more accurately calibrate coping effort to actual demand, producing better adjustment [12], [16], [23]–[25]. Second, physical symptoms, specifically dysphagia and pain level were hypothesized to detrimentally affect QoL. Eating dysfunction has been associated with poor QoL among patients with head and neck cancer and NPC [11], [14], [26], [27]. Pain is commonly reported by cancer patients and independently impairs QoL [28]. Third, we hypothesized that patient satisfaction with clinical services differentiates QoL trajectories. For instance, greater satisfaction with emotional and informational support from health care providers has been shown to predict better QoL among cancer patients [15]. Hence, we hypothesized that patients with little disruption of QoL would have high optimistic outlook, better satisfaction with emotional and informational support from health care providers, and low pain and eating dysfunction [16], [17], [20].

## Methods

**Figure 1:**
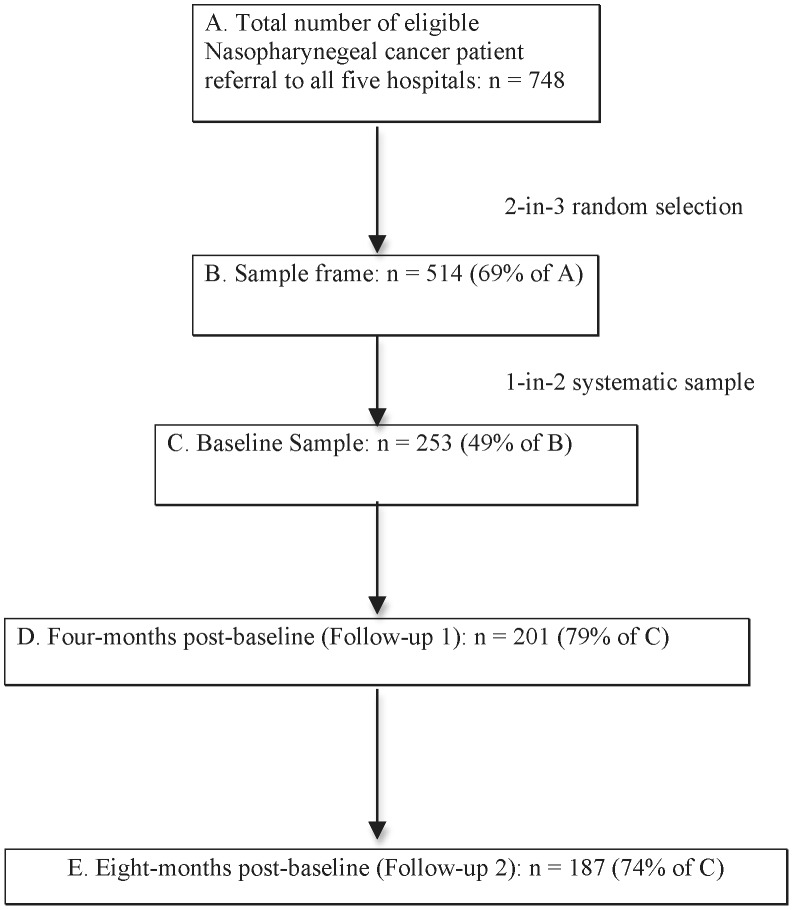
Sampling structure and attrition pattern of the study (13).

This secondary analysis was performed on the RADON dataset. Details of the RADON study recruitment are reported elsewhere [10]–[12]. Briefly, data were collected on a sample of NPC patients recruited before the onset of radiation therapy (RT) (Baseline) and again 4 (FU1) and 8 (FU2) months later (Figure 1). The aim of the original study was to examine the impact of RT on QoL among cancer patients. Hence, baseline aimed to assess pre-RT QoL status, FU 1 was chosen to assess the impact of active treatment on QoL, and FU 2 was chosen to assess patient's QoL status post-RT.

Following University of Hong Kong ethics committee approval, trained interviewers recruited patients from five different public regional hospital out-patient radiation oncology clinics across Hong Kong that handle over 90% of all NPC cases in Hong Kong. A mixed sampling strategy involved targeted clinic sessions in which all patients with a primary diagnosis of NPC who were attending for RT treatment planning were deemed eligible provided they could comprehend and complete the assessments and gave fully-informed written consent.

Interviews were performed in a private room at clinics and all assessments were administered orally using a standardized protocol involving response cards and validated instruments. Follow up interviews were also done face-to-face on return visits to the clinic, else telephone interviewing was used to assess patients for whom clinic interviews could not be arranged. Response categories were read out instead of being visually presented. A random sub-set of interviews were doubly coded by two interviewers simultaneously to assess inter-rater reliability and monitor inter-rater drift. All Kappa coefficients for these joint interviews remained above 0.9, indicating excellent reliability.

### Measures

#### i. Quality of life (QoL)

At the time of data collection (1996–1997) there was no specialized head and neck cancer subscale for any QoL instrument and no Chinese version of a QoL measure that had been validated. We therefore adopted the Functional Assessment of Cancer Therapy – General (FACT-G) (version 3) scale [29] and using an ethnographic translation procedure created and validated a Chinese version, the FACT-G (Ch) on a sample of 1,267 Hong Kong Chinese cancer patients [10]. The FACT-G (Ch) has good internal consistency (Cronbach's α 0.85). The convergent validity of the FACT-G (Ch) with a generic QoL measure (WHOQOL-BREF9(HK)) was 0.72 (*p*<0.001), and divergent validity supported by correlations below 0.15 with non-QoL measures. The original factor structure was valid in the study population though accountable factor variance was lower than in the original instrument [10]. The final FACT-G (Ch) did not use the doctor subscale from the original instrument due to low reliability, and has four subscales, measuring physical (Phy) (e.g. “I have a lack of energy”, “I have nausea.”), social/family (Soc/Fam) (e.g. I feel distant from my friends”, I get emotional support from my family”), emotional (Emt) (e.g. “I feel sad”, “I am proud of how I'm coping with my illness”), and functional well-being (Fnt) (i.e. “I am able to work”, “I am able to enjoy life”). Responses are scored 0–4 with higher scores equating with better QoL. The internal consistency of the subscales ranged from 0.53 to 0.75 [11]. The convergent validity of the subscales with the abbreviated version of World Health Organization QoL measure ranged from 0.33 to 0.65. Hence, the subscales of the FACT-G (Ch) demonstrated acceptable convergent validity and reliability. Total QoL scores are calculated by summing these subscales. We used these four subscales to assess trajectory patterns.

#### ii. Optimism

Optimism was assessed using a single-item visual analogue (VA) measure, with an 11-point (0–10) 10 cm line, labeled “0” and “10” at opposite ends [10], [28]. Participants were asked to rate the statement “My attitude to life in general is …” which was headed “completely pessimistic” “0” and “completely optimistic” “10”. The adoption of a single-item measure of optimism is not uncommon. Previous studies demonstrated a single-item measure of optimism was positively, moderately correlated with multiple items measure of optimism [30], [31]. Moreover, the item measuring optimism showed moderate inverse correlation with item measuring depression, further supporting the validity of this single-item trait measure [12].

#### iii. Dysphagia

Three dimensions of dysphagia were assessed including eating ability (“My eating ability is …”), eating appetite (“My eating appetite is …”), and eating enjoyment (“I enjoy eating …”) [14]. Each of these dimensions was assessed using a single-item VA 11-point scale, with the “0” end indicated “very bad”/“do not enjoy at all” and the “10” end indicated “very good”/“enjoy very much”. Within the RADON cohort, patients with NPC reported significantly greater dysphagia than patients with breast cancer, supporting the discriminate validity of these measures [14].

#### iv. Pain

Using a single-item VA scale patients were asked to rate their current pain (“Your pain level right now”) on an 11-point scale, with “0” indicating “No pain at all” and the “10” indicating “pain so severe as to prohibit all activity; the worst pain you can imagine” [28]. Physical symptom distress is a significant predictor of quality of life and pain is one of the most common physical symptom in cancer [32]. Pain is therefore expected to be associated with quality of life. The single-item measure of pain correlated negatively with quality of life on patients with lung cancer (Pearson correlation coefficient [r] = −0.52), suggesting acceptable construct validity [28].

#### v. Patient satisfaction

The revised Chinese validated version of Medical Information Satisfaction Scale [33] (C-MISS-R) patient's satisfaction with their medical consultation [34]. The five-item subscale measures the cognitive elements (understanding, expectations, and knowledge) of consultations [33]. Each item is scored on a five-point Likert scale from “strongly agree' to “strongly disagree.” Higher scores indicate greater satisfaction.

The Chinese Patient Satisfaction Questionnaire (ChPSQ-9) is an indigenous instrument for assessing patient satisfaction with Hong Kong outpatient clinical services [35]. The 9-item ChPSQ-9 assesses satisfaction with caring and supportive interactions on a 5-point Likert scale (1 = very dissatisfied, 2 = dissatisfied, 3 = OK, 4 = satisfied, 5 = very satisfied). Higher scores reflect greater satisfaction. The ChPSQ-9 has good internal consistency (Cronbach's α 0.93). The convergent validity of the ChPSQ-9 was indicated by its positive correlation with the Chinese Medical Information Satisfaction Scale-Revised (r = 0.27, p<0.01) [35].

#### vi. Mood

Since patients with negative affect are more likely to report poorer quality of life [36], we therefore adjusted for the effect of negative affect on QoL by including it as a confounding variable in multivariate analyses. For respondents this was labeled “Mood” and was assessed using a single item VA measure with an 11-point (0–10) 10 cm line, labeled “0” and “10” at opposite end. Participants were asked to rate statement “My mood is …”, which was headed “very bad” “0” and “very good” “10” [11]. The convergent validity of the single item measure of mood was demonstrated by its significant prediction of quality of life in a sample with mixed cancer types (p<.0001) [14].

#### vii. Demographic and clinical variables

Patients' socio-demographic data were collected at baseline interview, whereas clinical data were extracted from patients' medical record using a standardized form by a medically-qualified researcher following a standardized protocol. Disease stage was classified using Ho's staging classification for NPC [37], [38].

All measures were gathered at baseline excepting measures of eating function and pain, which were measured at FU1, and QoL, which was assessed at baseline, FU1, and FU2. Since both dysphagia and pain are common side-effects of cancer treatment including RT [5], [14], measures of dysphagia and pain were assessed at FU1 when patients were receiving active treatment.

### Data analysis

Standard descriptive analyses assessed sample characteristics. To examine patterns of Physical, Social/Family, Emotional, and Functional well-being over the eight months follow-up, we used a latent growth mixture model (LGMM) framework [39], derived using Mplus version 6.11. With longitudinal data LGMM tests whether the population under study comprises two or more discrete classes of individuals with differing profiles of growth (i.e. trajectories), with class membership determined by these different growth parameters. After determining the optimal number of component classes, examination of covariates can differentiate determinants or correlates of class membership. Mplus employs a robust full-information maximum-likelihood (FIML) estimation procedure for handling missing data. FIML assumes that missing data are unrelated to the outcome variable (missing at random) [40], [41].

Our analyses followed three steps [42]. First, we identified a univariate single-class growth model without covariates (the studied predictors). Secondly, we used fit indices to identify the optimal number of distinct trajectories without covariates. To optimize the number of trajectories, the Bayesian (BIC), sample-size adjusted Bayesian (SSBIC), and Aikaike information criteria (AIC), entropy values, the Lo-Mendell-Rubin likelihood test (LRT) and the bootstrap likelihood ratio test (BLRT) fit indices were used [42]. These criteria are recommended for determining the number of trajectories [42]. Moreover, we examined models in which the growth parameters and associated covariance were constrained to be equivalent across classes, and models in which these constraints were relaxed. We sought a model with lower values for the information criteria indices, higher entropy values, and p values≤0.05 for both the LRT and the BLRT. Thirdly, we extended the LGMM to include covariates of class membership in order to verify the correct model specification [42]. Because inclusion of too many covariates impairs model convergence, we only included the study predictors (pain, eating ability, eating enjoyment, eating appetite, optimism, ChPSQ-R, and C-MISS-R). Then, we used multinominal logistic regression to examine which, if any of the proposed predictors and confounding variables (including demographic, medical, and mood factors) differentiated trajectories [16], [17]. To assess the presence of multicollinearity, bivariate correlation analyses were conducted among proposed predictors [43], with correlations ≥0.9 suggesting substantial collinearity. Univariate analyses were initially used to assess the relationship between each of the confounding variables and trajectory patterns; only those significant associated with trajectory patterns were included in the multinominal logistic regression analyses. Lastly, the final multinominal logistic regression models retained only the significant study predictors and the potential confounding variables.

## Results

### Subject characteristics

At each clinic session every second patient in the sample frame was targeted for recruitment but if manpower shortages did not permit this, a 1 in 5 or 1 in 10 recruitment protocol was adopted as necessary. Of 748 new NPC patients attending the five hospitals during the data collection period, a 2-in-3 random selection produced 514 (69%) patients who formed the sample frame. Of these, 253 (49%) were recruited into the study using 1-in-2 systematic sampling. At FU1, 201 respondents were interviewed and at FU2 187, with an overall response rate of 79% and 74% respectively (Figure 1). Participants and dropouts differed significantly by cancer stage (χ^2^ = 10.532, p = .004), with dropouts comprising more patients with advanced disease (28.1% vs. 8.7%) [15]. Mean time between Baseline and FU1 was 3.8 months (S.D. 20.2 days) and between FU1 and FU2 was 3.9 months (s.d. 20.9 days). Telephone interviews were used for 27% of FU1 and 53% of FU2. Checks between telephone and face-to-face interview data revealed no significant differences in sample sociodemographic and clinical characteristics. Table 1 summarized baseline sample characteristics.

**Table 1:** Demographic and medical information of the sample (n = 253).

Characteristic	n (%)
Demographic information	
Age (years)	
Mean (SD)	48.81 (11.96)
Sex	
Male	181 (72)
Female	72 (28)
Marital status	
Single	26(10)
Married/Cohabited	209(83)
Divorced/Separated	8(3)
Widowed	10(4)
Education level	
No formal education	36 (14)
Primary education	96 (38)
Secondary education	95 (38)
Tertiary education	25 (10)
Family household income (per month)^1^	
≤HK$ 10,000	77 (30)
10,001–20,000	88(35)
20,001–30,000	27(11)
30,001–40,000	18(7)
Above 40,000	17(7)
Do not know	23(9)
No income	3(1)
Occupation	
Full-time employment	141(56)
Part-time employment	6 (2)
Retired/Housewife	75 (30)
Unemployed	30(12)
Years of Hong Kong residency	
≤7	7 (3)
>7	246 (97)
Medical information	
Cancer stage at diagnosis	
I	17 (7)
II	82(34)
III	114(47)
IV	23(10)
V	5(2)
Recurrence after baseline	
Yes	27(11)
No	219(89)
Treatment between baseline and FU1	
Yes	223(96)
No	10 (4)
Treatment between FU1 and FU2	
Yes	38(18)
No	177 (82)

SD: Standard deviation; FUI: Follow-up 1 (conducted 4 months after baseline); FU2: Follow-up 2 (conducted at 8 months after baseline).

1 $1 U.S. = $7.8 HK.

### Bivariate analyses of study variables

Table 2 shows correlation analyses between study variables. Most of the study predictors or potential confounders (age and mood) were either uncorrelated or correlated weakly, with the exception of the measures of dysphagia which were correlated moderately. Hence, no substantial collinearity was found among the study predictors, as well as the potential confounders.

**Table 2:** Correlation matrix of study variables.

	Appetite	Eating Enjoyment	Pain	C-MISS-R	ChPSQ-9	Optimism	Age	Mood
Eating ability	0.59**	0.50**	−0.37**	0.05	0.04	0.02	−0.11	0.11
Appetite		0.54**	−0.25**	0.01	0.11	0.01	−0.14*	0.14*
Eating Enjoyment			−0.24**	0.02	0.05	0.09	−0.06	0.09
Pain				0.12	0.01	−0.09	−0.06	−0.03
C-MISS-R					0.23**	0.14*	−0.11	0.23**
ChPSQ-9						0.09	0.09	0.17*
Age								−0.01

** p<.001;

* p<.05; C-MISS-R: The revised Chinese validated version of Medical Information Satisfaction Scale; ChPSQ-9: The Chinese Patient Satisfaction Questionnaire.

### Physical (Phy) functioning trajectories

#### i. Unconditional model

Preliminary analyses showed that the best fitting unconditional models were those in which variance for intercept and slope was constrained across classes. For Phy, the AIC, BIC, and SSBIC substantially decreased, showing progressively better fit in models of up to three classes (Table 3). LRT indicated a statistically insignificant difference between three-class and four-class models, further suggesting that the four-class model failed to improve fit [42].

#### ii. Conditional model

**Figure 2:**
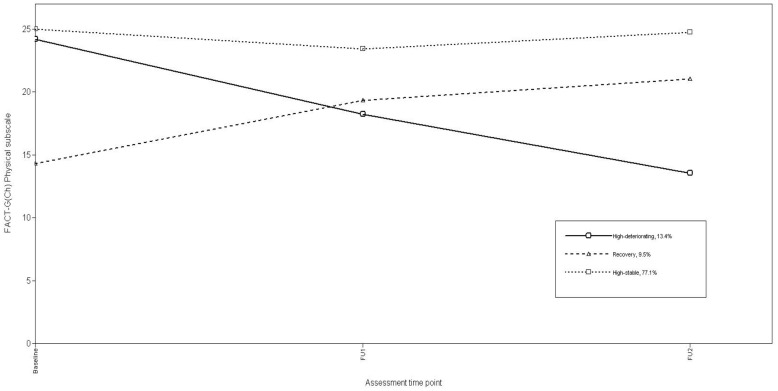
Trajectories of FACT-G(Ch) Physical subscale.

Using a three-class solution, we included the study predictors as specified above to specify a conditional model. Using log-likelihood ratio Chi-square (χ^2^) to assess the model fit, the conditional model with the study predictors significantly improved model fit (χ^2^(16) = 315.32, p<0.001). Growth parameter estimates for the final conditional model (Table 4) and associated trajectories for Physical functioning (Figure 2) assigned most patients (77.1%) to a “High-stable” trajectory characterized by largely unchanging, high Phy scores from Baseline to FU2. The second largest group (13.4%), of patients showed “High-deteriorating” trajectories featuring high initial Phy scores that steadily decreased over FU1 and FU2. The third group of patients (9.5%) followed a “Recovery” trajectory, having the lowest Phy scores at baseline, which subsequently improved over FU1 and FU2.

**Table 3:** Fit indices for selecting the number of trajectories.

No. of classes	AIC	BIC	SSBIC	Entropy	LRT p-value	BLRT p-value
Physical subscale						
1	4244.327	42172.594	4247.233			
2	4187.966	4216.233	4190.872	0.825	<.001	<.001
3	4143.305	4182.172	4147.300	0.861	.022	<.001
4	4138.213	4187.680	4143.298	0.857	.362	<.001
Emotional subscale						
1	3740.469	3768.736	3743.374			
2	3687.295	3715.562	3690.201	0.869	<.001	<.001
3	3662.271	3701.138	3666.266	0.887	.0950	<.001
4	3660.237	3713.238	3665.685	0.898	.1943	.0870
Social/family subscale						
1	3983.494	4011.762	3986.400			
2	3976.655	4004.952	3979.591	0.717	<.001	<.001
3	3973.555	4012.422	3977.550	0.618	.185	.065
4	3966.820	4016.287	3971.905	0.633	.013	<.001
5	3965.786	4025.853	3971.960	0.689	.036	.089
Functional subscale						
1	4418.907	4447.174	4421.813			
2	4407.791	4436.058	4410.697	0.649	<.001	<.001
3	4408.101	4445.244	4412.460	0.593	.0875	.333

AIC: Akaike Information criterion; BIC: Bayesian information criterion; SSBIC: sample size adjusted Bayesian information criterion; LRT: Lo-Mendell-Rubin Test; BLRT: bootstrap likelihood ratio test. Note: We sought a model with lower values for the information criteria indices, higher entropy values, and significant p values for both the LRT and the BLRT.

**Table 4:** Growth factor parameter estimates for three-class conditional models for FACT-G(Ch) subscales.

		Intercept			Linear Slope	
	Mean	SE	p-value	Mean	SE	p-value
Physical subscale# (Possible range 0–28) (Normative data: US general population mean 22.7 [44]; German general population mean 24.9 [45])						
High-deteriorating	23.926	0.613	<.001	−1.375	0.146	<.001
Recovery	13.843	1.317	<.001	0.896	0.204	<.001
High-Stable	24.561	0.268	<.001	−0.037	0.048	0.445
Emotional subscale# (Possible range 0–20) (Normative data: US general population mean 19.9 [44]; German general population mean 19.5 [45])						
High-stable	14.775	0.195	<.001	0.205	0.030	<.001
High-deteriorating	13.027	1.405	<.001	−0.543	0.315	0.084
Recovery	6.384	2.246	.004	0.835	0.362	0.021
Social/family subscale# (Possible range 0–28) (Normative data: US general population mean 19.1 [44]; German general population mean 20.2 [45])						
High-stable	22.071	0.500	<.001	0.159	0.085	.060
High-deteriorating	20.676	0.857	<.001	−0.368	0.118	.002
Recovery	15.543	1.633	<.001	0.995	0.141	<.001
Low-stable	14.051	1.093	<.001	0.308	0.210	0.143
Functional subscale# (Possible range 0–28) (Normative data: US general population mean 18.5 [44]; German general population mean 21.4 [45])						
High-stable	17.993	0.525	<.001	0.093	0.076	0.217
Low-decline	12.675	0.502	<.001	−0.266	0.131	0.043

SE, standard error;

# Higher scores indicate better quality of life.

#### iii. Differentiating Physical function trajectories

Phy trajectories were unrelated to age, martial status, occupation, education, household income, stage of disease, recurrence after baseline and mood. Gender was significantly correlated with Phy trajectories (χ^2^ = 7.48, p = 0.024). Consequently, multiple logistic regression compared study predictors by Phy trajectories, adjusted for gender. Only optimism and pain were retained (χ2 (4) = 29.55, p<.001). With High-stable trajectory as a reference, optimism, pain, and gender differentiated Phy trajectories, accounting for 12% of variation in class status (Cox and Snell R^2^). Compared with the High-stable trajectory patients, Recovery trajectory patients reported significantly greater pain (odds ratio (OR) 1.53, 95% confidence interval (95% CI) 1.25–1.86) and less optimism (OR 0.78, 95% CI 0.63–0.97), whereas High-deteriorating trajectory patients had greater pain (OR 1.32, 95% CI 1.11–1.56) (Table 5).

**Table 5:** Multinomial logistic regression of predictors on QoL trajectories (High-stable group as referent).

	Physical subscale		
Predictors	Odds ratio (95% CI)	SE	P value
“High-deteriorating” group			
Pain	1.318(1.112–1.562)	.087	.001
Optimism	1.047 (.872–1.257)	.093	NS
“Recovery” group			
Pain	1.527(1.253–1.862)	.101	<.001
Optimism	.781 (.628–.971)	.111	.026

	Emotional subscale		
Predictors	Odds ratio (95% CI)	SE	P value
“High-deteriorating” group			
Pain	0. 958(.716–1.282)	.149	NS
Eating enjoyment	.778(.641–.945)	.099	.012
Optimism	1.007 (.788–1.287)	.125	NS
Ch-MISS-R	.879 (.779–.992)	.062	.037
Gender (Female as a reference)	.222(.078–.635)	.536	.005
“Recovery” group			
Pain	1.551(1.175–2.047)	.142	.002
Eating enjoyment	.758(.570–1.009)	.146	NS
Optimism	.618 (.444–.859)	.168	.004
Ch-MISS-R	.899 (.765–1.055)	.082	NS
Gender (Female as a reference)	.190 (.044–.823)	.747	.026

	Social/family subscale		
Predictors	Odds ratio (95% CI)	SE	P value
“High-deteriorating” group			
Age	1.016(.984–1.050)	.017	NS
Appetite	.961(.819–1.128)	.082	NS
Optimism	.985(.823–1.180)	.092	NS
Marital status (Married as a reference)	3.501(1.182–10.372)	.554	.024
Family income ≤$10,000 (Income >$20,000 as a reference)	1.154(.417–3.196)	.520	NS
Family income $10,0001–20,000 (Income >$20,000 as a reference)	1.246(.534–2.907)	.432	NS
“Recovery” group			
Age	1.080(1.031–1.131)	.024	.001
Appetite	.906 (.723–1.136)	.115	NS
Optimism	.757(.597–.961)	.121	.022
Marital status (Married as a reference)	7.194(1.786–28.970)	.711	.005
Family income ≤$10,000 (Income >$20,000 as a reference)	4.136(.910–18.793)	.772	NS
Family income $10,0001–20,000 (Income >$20,000 as a reference)	1.808(.391–8.354)	.781	NS
“Low-stable” group			
Age	1.030(.986–1.075)	.022	NS
Appetite	.800(.644–.992)	.110	.042
Optimism	.696(.555–.874)	.116	.002
Marital status (Married as a reference)	9,958(2.589–38.296)	.687	.001
Family income ≤$10,000 (Income >$20,000 as a reference)	9.876(2.212–44.099)	.763	.003
Family income $10,0001–20,000 (Income >$20,000 as a reference)	1.053(.196–5.665)	.859	NS

	Functional subscale		
Predictors	Odds ratio (95% CI)	SE	P value
“Low-decline” group			
Family income ≤$10,000 (Income >$20,000 as a reference)	4.228(1.670–10.701)	.474	.002
Family income $10,0001–20,000 (Income >$20,000 as a reference)	1.285(.532–3.105)	.450	NS
Eating enjoyment	.754 (.665–.855)	.064	<.001
Optimism	.772 (.656–.908)	.083	.002
Ch-MISS-R	.881 (.811–.958)	.042	.003

### Emotional (Emt) functioning trajectories

#### i. Unconditional model

The best fitting unconditional models were those in which variance for intercept and slope was constrained across classes. Fit statistics suggested the best-fitting model for Emt was a 3-class model (Table 3).

#### ii. Conditional model

**Figure 3:**
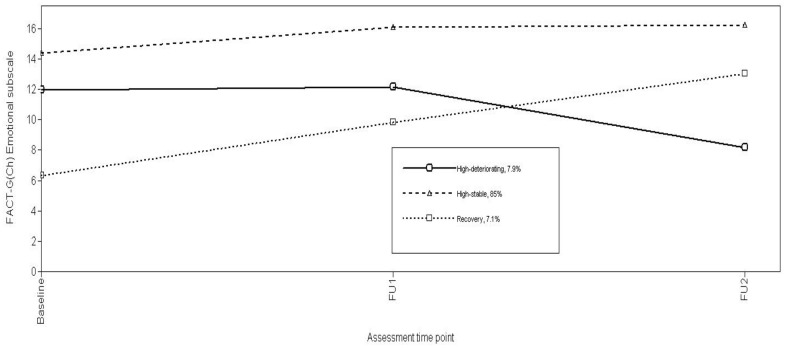
Trajectories of FACT-G(Ch) Emotional subscale.

Using log-likelihood ratio χ^2^, the conditional model with the study predictors significantly improved model fit (χ^2^(28) = 796.52, p<0.001). Growth parameter estimates for the final conditional model (Table 4) and associated Emt trajectories (Figure 3) classed most patients (85%) in a High-stable trajectory featuring high Emt scores at all assessment points. The remaining patients were evenly distributed between High-deteriorating (7.9%) or Recovery (7.1%) groups characterized by comparable trajectories described above for Physical functioning.

#### iii. Differentiating Emotional functioning trajectories

Emt trajectories were unrelated to age, martial status, occupation, education, household income, stage of disease, recurrence after baseline and mood. Gender was significantly correlated with Emt trajectories (χ^2^ = 7.99, p = 0.018). Consequently, multiple logistic regression compared study predictors by Emotional trajectories, adjusted for gender. Optimism, pain, eating enjoyment, medical information satisfaction, and gender were retained (χ2 (10) = 49.647, p<.001), accounting for 21% of variation in class status (Cox and Snell R^2^). Compared to High-stable trajectory patients, those in the High-deteriorating and Recovery trajectory groups were less likely to be male (OR 0.22, 95% 0.08–0.63; OR 0.19, 95% CI 0.04–0.82, respectively). High-deteriorating trajectory patients also reported poor Eating enjoyment (OR 0.78, 95% CI 0.64–0.94) and less satisfaction with medical information (OR 0.88, 95% CI 0.78–0.99), whereas Recovery trajectory patients had higher pain (OR 1.55, 95% CI 1.17–2.05) and less optimism (OR 0.62, 95% CI 0.44–0.86) (Table 5).

### Social/Family (Soc/Fam) functioning trajectories

#### i. Unconditional model

The best fitting unconditional models were those in which variance for intercept and slope was constrained across classes. Fit statistics suggested the best-fitting model for Social/Family functioning was a 4-class model (Table 3).

#### ii. Conditional model

**Figure 4:**
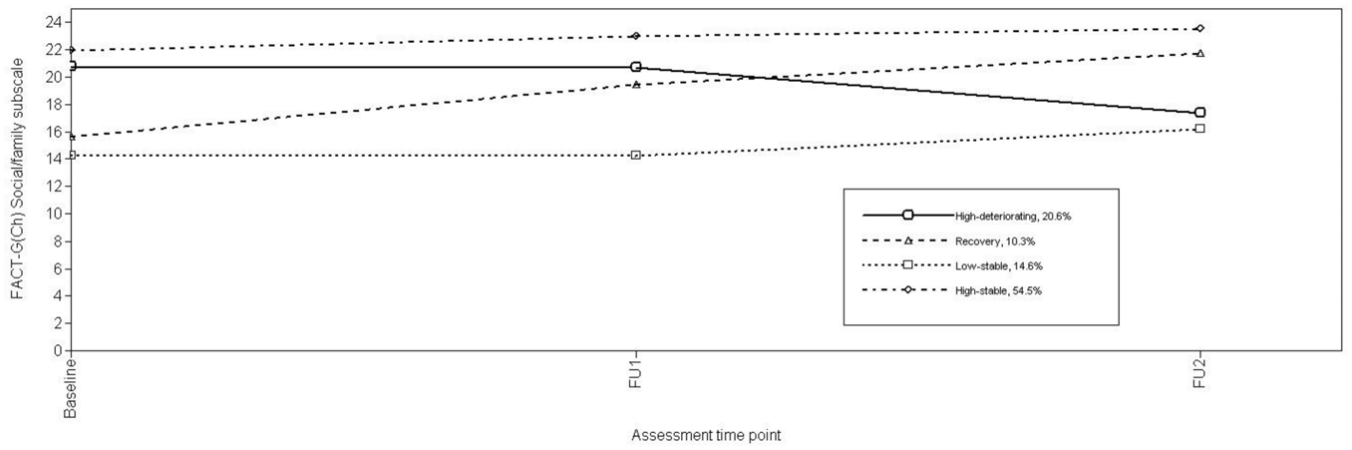
Trajectories of FACT-G(Ch) Social/Family subscale.

Using log-likelihood ratio χ^2^, the conditional model with the study predictors significantly improved model fit (χ^2^(20) = 413.01, p<0.001). Growth parameter estimates for the final conditional model (Table 4) and associated Soc/Fam trajectories (Figure 4) identified 54.5% of patients as evidencing a High-stable Soc/Fam score trajectory over time. One fifth (20.6%) of patients demonstrated High-deteriorating trajectories in Soc/Fam scores being high at Baseline, but declining thereafter. “Low-stable” (14.6%) (Soc/Fam scores from Baseline to FU2), and “Recovery” (10.3%) (gradually improving Soc/Fam scores from Baseline to FU2) trajectories classified the remaining patients.

#### iii. Differentiating Social/Family functioning trajectories

Soc/Fam trajectories were unrelated to occupation, gender, stage of disease, recurrence after baseline and mood, but correlated with age (F = 7.913, p<.001), martial status (χ^2^ = 31.69, p<0.001), education (χ^2^ = 9.28, p = 0.026), and household income (χ^2^ = 41.31, p<.001). Consequently, multiple logistic regression compared study predictors by Soc/Fam trajectories, adjusted for age, martial status, education, and household income. Optimism, eating appetite, age, household income, and martial status were retained (χ2 (18) = 74.59, p<.001), accounting for 31% of variation in class status (Cox and Snell R^2^).. Compared to High-stable trajectory patients, High-deteriorating, Recovery, and Low-stable trajectory patients were more likely to be single/divorced/widowed (OR 3.50, 95% CI 1.18–10.37; OR 7.19, 95% CI 1.79–28.97; OR 9.94, 95% CI 2.59–38.30, respectively). Recovery trajectory patients also were older (OR 1.08, 95% CI 1.03–1.13). Low-stable trajectory patients had poor eating appetite (OR 0.80, 95%CI 0.64–0.99) and lower household incomes (OR 9.88, 95% CI 2.21–44.1). Recovery and Low-stable trajectory patients were more likely to report less optimism (OR 0.76, 95% CI 0.60–0.96; OR 0.70, 95% CI 0.55–0.87, respectively) (Table 5).

### Functional (Fnt) well-being trajectories

#### i. Unconditional model

The best fitting unconditional models were again those in which variance for intercept and slope was constrained across classes. Fit statistics suggested the best-fitting model for Fnt was a 2-class model (Table 3).

#### ii. Conditional model

**Figure 5:**
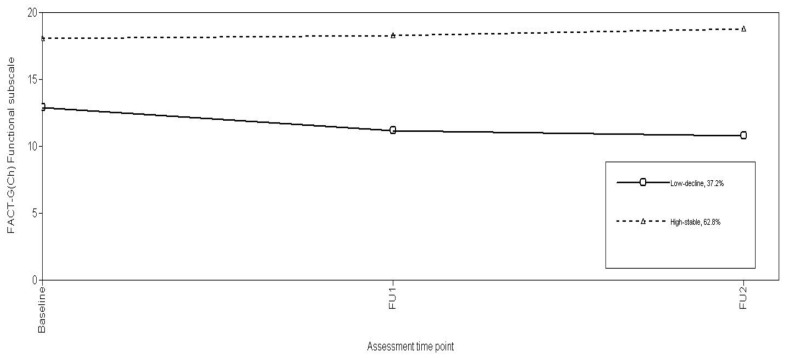
Trajectories of FACT-G(Ch) Functional subscale.

Using the log-likelihood ratio χ^2^, the conditional model using the study predictors significantly improved model fit (χ^2^(21) = 621.07, p<0.001). Growth parameter estimates for the final conditional model (Table 4) and Fnt trajectories (Figure 5) indicate most patients (62.8%) evidenced a “High-stable” trajectory over time. The remaining patients displayed “Low-decline” (37.2%) trajectories characterized by low Functional well-being scores at Baseline, which further declined over FU1 and FU2.

#### iii. Differentiating Functional well-being trajectories

Fnt trajectories were unrelated to marital status, gender, stage of disease, recurrence after baseline but correlated with age (t = 2.37, p = .019), mood (t = −3.67, p<.001), occupation (χ^2^ = 7.54, p = 0.023), education (χ^2^ = 5.22, p = 0.022), and household income (χ^2^ = 17.27, p<.001). Consequently, multiple logistic regression compared study predictors by Soc/Fam trajectories, adjusted for age, mood, martial status, education, and household income. Optimism, eating enjoyment, age, medical information satisfaction, and household income were retained (χ2 (5) = 58.26, p<.001), accounting for 26% of variation in class status (Cox and Snell R^2^). Compared to the High-stable trajectory patients, Low-decline trajectory patients reported poor Eating enjoyment (OR .75, 95% CI .66–.85), and reported lower satisfaction with medical information (OR 0.88, 95% CI 0.81–0.96), lower optimism (OR 0.77, 95% CI 0.66–0.91) and they more likely from lower family household income (OR 4.23, 95% CI 1.67–10.7) (Table 5).

## Discussion

This analysis described trajectories of change in four QoL domains (physical, emotional, social/family, and functional well-being) of the FACT-G(Ch) over 8 months before, during and after radiation therapy for NPC. While previous studies using averaged data showed QoL improves progressively throughout the first year following the diagnosis of NPC [10], [13], our LGMM analyses shows that this is largely incorrect and obscures important sub-group differences in adaptation. Most patients experienced stable high levels of emotional (85%), physical (77%), functional (63%) and social/family (55%) well-being during the 8-month RT period. This agrees with earlier studies exploring distinct change trajectories in adjustment during early breast cancer [17], [20], [22] and advanced cancer [19], adding to the growing evidence that most patients have little or no significant disruption of QoL functioning during cancer. This is an important message to communicate to both clinicians and patients, many of whom are highly anxious about cancer treatments; that they are well coped with by many patients can provide a powerful reassurance that new patients too can survive cancer treatments.

Different trajectory patterns occur across the four QoL domains; three for physical and for emotional well-being, four for social/family well-being, and two for functional well-being. We believe this is the first time these trajectories for different QoL domains have been described. Previous studies explored psychological distress/well-being with some including physical [20] and spiritual well-being [19]. Our study offers new insights into patterns of social/family and functional well-being not previously addressed and caries important implications for the targeting and provision of support services for cancer patients.

In both physical and emotional domains, most NPC patients conformed to High-stable trajectories, maintaining average scores at each assessment point of ≥75% of possible maximum scores. Because there is no available normative data on FACT-G for the general Hong Kong or Chinese adult population, a post-hoc comparison of the QoL scores in our sample with normative data for the general US adult population [44], and general German adult population [45] (Table 4) shows the High-stable trajectory patients demonstrated QoL scores comparable with normative data for the general US and German adult populations. These stable high levels of physical and emotional well-being over time contrast markedly with the fractions of patients (13% vs. 8% respectively) perceiving deterioration in physical and emotional well-being during treatment. Patients with High deteriorating trajectories showed sustained declines over time from levels comparable to High stable patients, while emotional well-being was high until FU1 when patients were receiving radiation therapy and declined thereafter. Only 10% and 7% of patients demonstrated the classical “recovery” trajectory of physical and psychological adjustment respectively, from initial poor functioning that gradually improved. Our analyses did not show evidence of trajectory patterns consistent with persistent disruption that have been consistently reported in previous studies on women with breast cancer [17], [20], [22]. Because of assessment similarities, this may imply that different cancer sites and types generate more or less impact, or because QoL impacts differ from, or different mechanisms pertain to psychological distress.

Social/family well-being showed four trajectories. In addition to the High-stable, High-deteriorating, and Recovery trajectories described in the previous paragraph, the Low-stable trajectory characterized by persistent low social/family well-being over time included the ∼15% of NPC patients reporting the lowest scores throughout the study. The mean scores of the Low-stable trajectory over time (mean 13.92 at baseline, 14.27 at FU1, and 16.68 at FU2) were consistently well below the normative data from the US (mean 19.1) and German populations (mean 20.2). Functional well-being evidenced just two trajectories; High-stable and Low-decline, a pattern not seen for other domains, where a significant proportion of patients reported low functional well-being at baseline that declined further over time, probably reflecting treatment side-effects on function. Again, the mean scores of the Low-decline trajectory over time (mean 12.47 at baseline, 10.84 at FU1, 10.30 at FU2) were lower than the normative data from the US (mean 18.5) and German (21.4) populations.

Of interest is what differentiated these QoL domain trajectories. Excepting optimism, which differentiated trajectories in all four domains, different variables separated the trajectory groups within domains. Respondents with the initial lowest level of well-being reported a less optimistic outlook than did those in the High-stable group in all domains. This concurs with prior studies, suggesting that patients holding negative future expectations perhaps are more likely to adopt ineffective coping strategies when facing cancer diagnosis thereby impairing adjustment [17], [25], [46], [47].

An inverse relationship between pain and QoL has been reported [28], [48]. However in this study pain during RT only differentiated physical and emotional well-being trajectories. For physical well-being, High-stable trajectory patients reported significantly less pain during RT than patients in the High-deteriorating or Recovery groups. For emotional well-being, patients in the Recovery trajectories reported more pain during RT than patients in high-stable group. The Phy domain includes one item assessing pain and this might have lead to inflated estimates for this domain, but the same pattern is seen for Emt which includes no pain item, and for both QoL domains the same Recovery trajectory was characterized partly by greater pain reports.

Aspects of dysphagia differentiated trajectories in emotional well-being, social/family well-being, and functional well-being. Eating enjoyment differentiated trajectories in emotional and in functional well-being, whereas eating appetite differentiated social/family well-being trajectory. In the emotional well-being domain, eating enjoyment differentiated High-stable from High-deteriorating trajectory patients. Patients following the High-deteriorating trajectories reported less eating enjoyment than those on the High-stable trajectories. Similarly, patients in the functional Low-decline trajectory reported less eating enjoyment than did those in the High-stable trajectory. In the family/social well-being domain, only eating appetite differentiated High-stable from Low-stable trajectories, with Low-stable trajectory patients reporting less eating appetite than High-stable trajectory patients. Swallowing dysfunction and hyposalivation are two common side-effects of RT for patients with NPC [5], [13], [49]. It is not surprising therefore that dysphagia negatively influences QoL. Our findings highlight how different dimensions of eating play unique roles in influencing different domains in QoL. Poor appetite affects family and social relationships particularly in Chinese families where communal meals are important social events [50], whereas loss of eating enjoyment can negatively affect emotion and reflect impaired daily function. Hence, effective oral care during RT treatment is essential to prevent detrimental effects on QoL.

Satisfaction with cognitive elements of consultation predicted trajectories in emotional well-being and functional well-being domains. Within the functional well-being domain, High-stable trajectory patients reported greater satisfaction with cognitive dimensions of medical consultation than did those in the Low-decline trajectory. Within the emotional well-being domain, satisfaction with cognitive aspects of medical consultation differentiated patients in the High-stable trajectory from those in the High-deteriorating group, with the former reporting greater consultation satisfaction. Cognitive aspects of medical consultation focus on information adequacy. Perhaps poor communication of treatment side-effects and prolonged functional impairments and residual symptoms affecting eating also lead to later emotional distress as the permanent nature of the impairments became apparent. This is consistent with a previous trajectory study wherein women reporting greater dissatisfaction with the medical consultation showed delayed psychological distress after the diagnosis of breast cancer [16].

Except for the physical well-being domain, demographic factors were significant in differentiating trajectories of QoL domains. Sex predicted trajectories in emotional well-being. Compared to High-stable patients, High-deteriorating or Recovery group patients were more likely to be female. Our findings concur with previous QoL studies where gender [51] was associated with QoL. Family household income predicted trajectories in functional well-being, with Low-decline trajectory patients more likely to have the lowest level of family monthly income. Most families in Hong Kong employ a domestic helper to take care of the home as most adults work full time. Our findings may highlight the need for support in daily activities among patients from the lower socio-economic class who are probably unable to afford to hire a domestic helper to do the daily chores. Within the social/family well-being domain, patients who were married were more likely to be in the High-stable trajectory, suggesting patients who were single are at risk from inadequate social support. Chinese patients tend to hide their cancer diagnosis from friends due to the fear of stigmatization [52]. Previous studies on Chinese women with breast cancer reported women who did not disclose their diagnosis to friends consistently reported poorer social functioning [53]. However, we did not assess whether patients in the current study had disclosed their diagnosis to friends and therefore, we cannot assess its influence on social well-being.

Several limitations to this manuscript exist. We assessed initial QoL at the pre-RT stage but were unable to assess QoL at the diagnostic stage which prevented us from examining if QoL at diagnosis, or indeed diagnosis itself, affects the subsequent QoL trajectories. With only three assessment time points, we were constrained to assess only linear changes over time for the overall group; this may have influenced the sub-group trajectories. Because we only followed patients up to 8 months how the patterns of QoL continue to unfold is unknown. To minimize assessment burden and facilitate retention in the study, most of the measures for the proposed predictors were single-item measures leading to the limited content validity as well as scale stability. Lastly, a relatively small sample size may have limited the ability to detect low frequency trajectories and therefore may not completely reflect all the potential subgroups.

In summary, few decrements in QoL over the eight months following the RT were observed in these patients treated for NPC. This has important implications for support service provision. Optimism plays an important role in maintaining QoL. Interventions helping patients to achieve and maintain an optimistic view should be implemented at the initial diagnostic stage. Different symptoms impact on different QoL domains and highlight the importance of assessing how each of the symptoms uniquely affects various dimensions of QoL. In particular, information satisfaction prevents decrement in emotional and functional well-being, reflecting the importance in helping patients to establish a realistic expectation of treatment impacts, which is reliant on high quality communications. Supportive care in daily activities is important for patients with lower socio-economic status, whereas social support may be more important for patients who are single.
